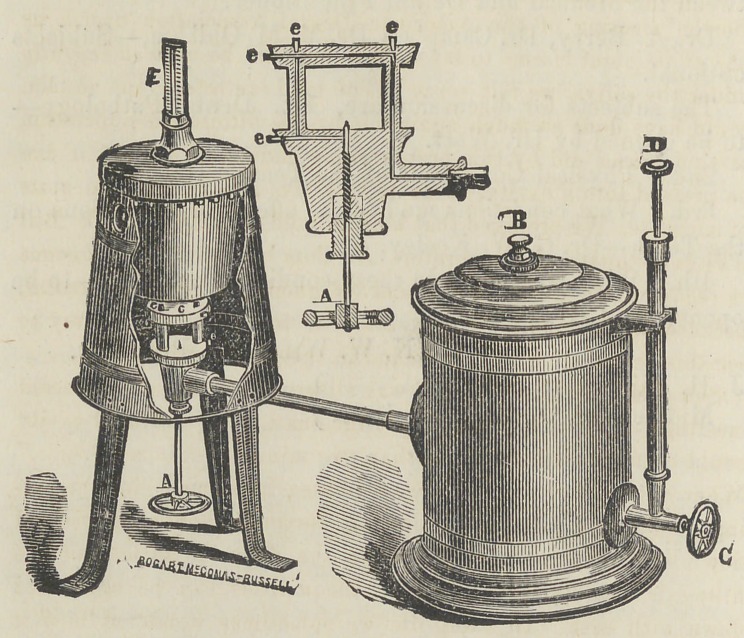# Hull’s Benzine Gas Burner

**Published:** 1867-08

**Authors:** 


### Editorial.

### HULL'S BENZINE GAS BURNER.

The accompanying cut represents a self-acting lamp for Dental purposes, and indeed for many other uses; invented by Mr. Hull, of this city. The Benzine is converted into gas in the burner, which is driven out by compressed air upon the fluid in the lamp. The air is forced in by the air pump, D C. This lamp is very convenient and efficient; it makes an intense heat, and is far more economical than alcohol, oil or ordinary gas.

The heat is under perfect control, increasing or diminishing by turning the valve wheel A. It is just the thing for soldering, melting, vulcanizing, etc.

Two quarts of fluid may be put into the lamp at once; and the blast will be sustained till it is all consumed, which will occupy from ten to twelve hours.

It should be in the Laboratory of every Dentist.

## Figures and Tables

**Figure f1:**